# Treatment with dopamine β-hydroxylase (DBH) inhibitors prevents morphine use and relapse-like behavior in rats

**DOI:** 10.1007/s43440-021-00307-2

**Published:** 2021-07-08

**Authors:** Małgorzata Frankowska, Paulina Surówka, Agata Suder, Renata Pieniążek, Renata Pukło, Joanna Jastrzębska, Władysława A. Daniel, Małgorzata Filip, Magdalena Zadrożny-Bujalska, Patrycja Kleczkowska

**Affiliations:** 1 Department of Drug Addiction Pharmacology, Maj Institute of Pharmacology, Polish Academy of Sciences, ul. Smętna 12, 31-343 Kraków, Poland; 2 Department of Pharmacokinetics and Drug Metabolism, Maj Institute of Pharmacology, Polish Academy of Sciences, ul. Smętna 12, 31-343 Kraków, Poland; 3 Department of Department of Pharmacodynamics, Centre for Preclinical Research and Technology, Medical University of Warsaw, ul. Banacha 1B, 02-097 Warsaw, Poland; 4 Military Institute of Hygiene and Epidemiology, ul. Kozielska 4, 01-163 Warsaw, Poland

**Keywords:** Disulfiram, Locomotor activity, Microdialysis, Morphine self-administration, Nepicastat, Rats, Seeking-behavior

## Abstract

**Background:**

Opioid use disorders are serious contributors to the harms associated with the drug use. Unfortunately, therapeutic interventions for opioid addicts after detoxification have been limited and not sufficiently effective. Recently, several studies have led to promising results with disulfiram (DSF), a dopamine β-hydroxylase (DBH) inhibitor, showing that it is a potent agent against not only alcohol but also addiction to various drugs.

**Materials and methods:**

This study was designed to examine whether DSF and nepicastat (NEP; another DBH inhibitor) modify morphine intake and reinstatement of seeking-behavior using the rat model of intravenous morphine self-administration. Additionally, we intended to estimate the effects of both inhibitors on the locomotor activity as well as on extracellular dopamine and its metabolite levels in the nucleus accumbens using microdialysis in naive rats.

**Results:**

We demonstrated that both DBH inhibitors reduced responding to morphine self-administration. Moreover, DSF and NEP administered acutely before reinstatement test sessions consistently attenuated the reinforcing effects of morphine and a morphine-associated conditioned cue. The observed effects for lower doses (6.25–25 mg/kg; *ip*) of both DBH inhibitors seem to be independent of locomotor activity reduction and dopamine level in the nucleus accumbens. Neither DSF nor NEP administered daily during morphine abstinence with extinction training sessions had any effect on active lever-responding and changed the reinstatement induced by morphine priming doses. Reinstatement of drug-seeking behavior induced by a conditioned cue previously associated with morphine delivery was attenuated following repeated administration of DSF or NEP during the abstinence period.

**Conclusion:**

These results seem to point to the significance  of DBH inhibition as a potential pharmacotherapy against morphine use disorders.

**Graphic abstract:**

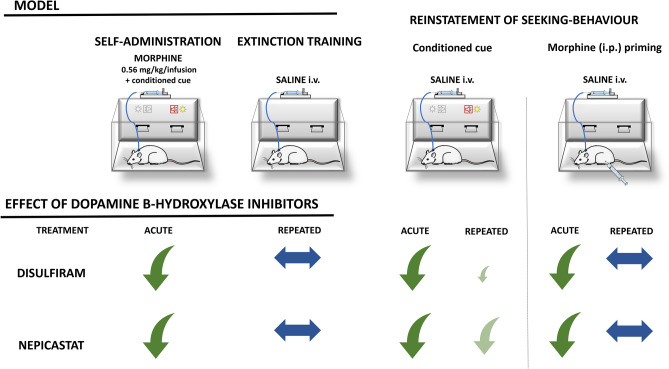

## Introduction

According to the Department of Mental Health and Substance Abuse (World Health Organization) as well as the European Monitoring Center for Drugs and Drug Addiction, the opioid use disorders (OUD), including compulsive using of pain relievers (e.g. morphine, tramadol), heroin, and synthetic opioids, are serious contributors to the harms associated with the drug use in the world [1]. The beginnings of the opioid overuse epidemic date back to the mid-1980s, when shifts in clinical practice planted the seed for the sharp uptick in prescribing opioids. During the last 10 years, an increasing trend in drug-related acute emergency cases was observed (c.a. 60%), particularly those caused by opioids and opiates. Moreover, OUD represents the most common form of addiction among entrants to specialized treatment [1, 2]. At present, unfortunately, the effectiveness of therapeutic interventions for opioid addicts after detoxification has been limited only to a substitution therapy [3].

On the whole, the knowledge about the role of dopamine in addiction, as the primary mediator of drug reward properties, has been solidified [4]. Thus, the drugs of abuse induced a large release of dopamine in limbic areas, specifically nucleus accumbens [4, 5]. The experimental results from the research into the role of dopamine as a primary neurotransmitter in OUD have been fraught with disagreement [6, 7]. The first evidence of the importance of another catecholamine, noradrenaline, in the mediation of OUD was emphasized at the beginning of the ‘70 s of the last century. For example, a series of experiments showed that AMPT, a tyrosine hydroxylase inhibitor, attenuated self-administration of morphine in rodents [8] and nonhuman primates [9] and that the mechanism was linked to the reduction of noradrenaline and dopamine synthesis. Moreover, U-14,624 or FLA-57, dopamine *β*-hydroxylase (DBH) inhibitors that convert dopamine into noradrenaline in noradrenergic neurons, attenuated the oral intake of morphine in rats [10, 11]. Recent studies demonstrated that other DBH inhibitors, like disulfiram (DSF) or nepicastat (NEP), effectively attenuated reinstatement of cocaine-seeking behavior in rats [12–15]. DSF, apart from inhibition of aldehyde dehydrogenase that results in acetaldehyde accumulation upon alcohol ingestion, also inhibits the DBH enzyme [16, 17]. Compared to DSF, NEP demonstrates significantly higher potency to inhibit DBH, without exerting the effect on other enzymes [18].  Several of studies revealed that both DBH inhibitors administered acutely reduced noradrenaline levels in the prefrontal cortex and nucleus accumbens, and effectively increased dopamine release only in the prefrontal cortex, without the influence on the nucleus accumbens [15, 19, 20].

Alcohol and opioids are commonly used together, while alcohol contributes to many opioid overdose deaths. The last recommendations for alcohol use disorder treatment present the use of naltrexone, acamprosate, and DSF as the first-line treatments for patients with alcohol use disorder, while the standard treatment for OUD proposes methadone, buprenorphine, and naltrexone [21–23]. Nevertheless, current clinical trials do not advise using inhibitors of DBH in the comorbidity of alcohol and OUD.

This study was designed to examine whether DSF and NEP modify behavioral responses to morphine and reinstatement of the drug-seeking-behavior using an animal model of intravenous self-administration. Importantly, to broaden our understanding of the mechanism of action of both above DBH inhibitors, we assessed the effects of acute and repeated treatment with DSF and NEP on locomotor activity and on extracellular dopamine release and its metabolite levels in the nucleus accumbens using microdialysis in freely moving naive rats.

## Materials and methods

### Animals

Experimentally naive male Wistar rats (225–250 g) delivered by a licensed breeder (Charles River, Munich, Germany) were housed in standard laboratory conditions (22 ± 2 °C temperature; 45–65% humidity; a 12-h light–dark cycle with lights on at 6.00 a.m.). Animals had free access to food and water during the 7-day habituation period (unless initial training sessions, see below). All experiments were conducted during the light phase of the light–dark cycle (between 7.00 a.m. and 4.00 p.m.). The experiments were carried out in accordance with the European Directive 2010/63/EU and were approved by the Ethical Committee at Maj Institute of Pharmacology, Polish Academy of Sciences, Kraków (permits no.: 1190/2015 and 336/2017).

### Drugs

Morphine (Polfa Warszawa, Poland) was dissolved in sterile 0.9% NaCl and given intravenously (0.1 ml/infusion) or intraperitoneally (*ip*; 1 ml/kg). Disulfiram (DSF; *N,N,N',N*-tetraethylthiuram disulfide, Polfa Warszawa, Warszawa, Poland) suspended in 0.1% methylcellulose (Sigma Aldrich, St. Louis, MO, USA) and nepicastat (NEP, BOC Sciences New Yorke, NY, USA) suspended in saline with 1.5% (v/v) DMSO (Sigma Aldrich, St. Louis, MO, USA) and 1.5% ethanol (POCH, Gliwice, Poland) were pre-administered 120 min, *ip* (1 ml/kg). Doses and pre-administration time of both DBH inhibitors were chosen based on previous research [13, 14, 24].

### Behavioral experiments

#### Morphine self-administration and extinction training

After a week of habituation to the animal facility, all animals were trained to press the lever for water reinforcement on a fixed ratio (FR) 1 schedule of reinforcement in standard operant chambers (Med-Associates, St. Albans, VT, USA). After the lever-press training and after giving the animals free access to water, rats were chronically implanted with a silastic catheter in the external jugular vein. Deep anesthesia was produced by a combination of ketamine hydrochloride (75 mg/kg *ip*; Bioketan; Biowet, Pulawy, Poland) and xylazine (5 mg/kg *ip*; Sedazin; Biowet, Pulawy, Poland). Following surgery, rats were kept individually in standard rat home cages with free access to water and food and catheters were flushed every day (0.1 ml heparin (70 U/ml) with saline solution or cephazolin solution (10 mg/ml; Biochemie GmbH, Kundl, Austria). Morphine self-administration testing began at least 7 days following surgery and was conducted in experimental chambers (Med-Associates, St. Albans, VT, USA) during 2-h daily sessions performed 6 days/week (maintenance). The infusion of morphine was associated with a conditioned stimulus (5 s), a tone (2000 Hz; 15 dB) and the illumination of the stimulus light directly above the active lever. Following each drug injection, there was a 20 s time-out period during which the response was recorded but had no programmed consequences. Response on the inactive lever never resulted in morphine delivery.

Initial training for morphine self-administration consisted of daily sessions during which each active lever press was reinforced with a morphine infusion at a dose of 0.56 mg/kg according to the FR1 schedule of reinforcement and the following acquisition. After 3 sessions of morphine self-administration, the response requirements were increased to FR3, then to FR5 for another 3 sessions, and were maintained till the end of the experiment. Animals encountering problems with the catheters during the self-administration period or rats that did not fulfill the self-administration acquisition/maintenance criterion (see below) were excluded from further experiments.

After morphine self-administration (at least 20 days), once the rats met the acquisition/maintenance criterion—that is (i) pressing the active lever for 3 successive days with 15% or less variation, on average and (ii) total intake of 90 mg morphine during the entire experiment—separate groups of rats underwent extinction training and reinstatement tests. The extinction sessions took place in experimental cages and covered 2-h daily training sessions neither with morphine provision nor exposure to a conditioned stimulus. After extinction training, covering not less than 14-daily sessions, the number of responses on the active lever fell below 15%, in comparison to the number of activations of that lever scored at the maintenance phase of morphine self-administration. Animals that failed to achieve an extinction training criterion were removed from experiments in reinstatement seeking-behavior. The animals underwent trial for the response reinstatement triggered by a non-contingent morphine injection or a conditioned cue (light + tone). The latter one was earlier associated with morphine self-administration. During 2-h reinstatement sessions active lever responding was rewarded with an intravenous saline injection.

##### Pharmacological tests

*Experiment 1: Morphine self-administration, extinction training, and reinstatement of drug-seeking behavior*

**Fig. 1:**
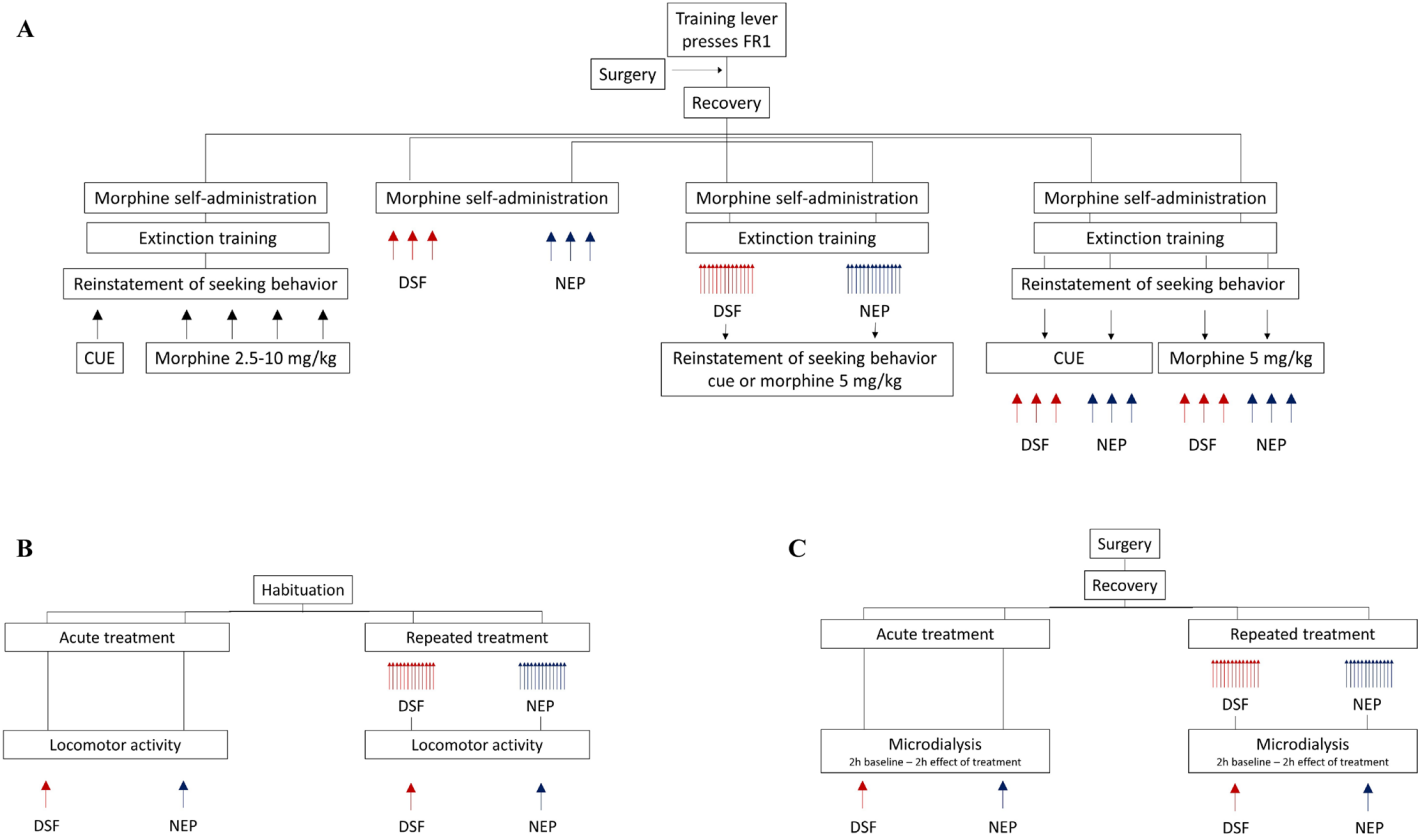
Experimental design of behavioral experiments including morphine self-administration, extinction training and cue- or morphine-induced reinstatement of seeking behavior (**A**), locomotor activity (**B**) or microdialysis procedures (**C**) and effects of disulfiram (DSF) or nepicastat (NEP). Each line represents a separate group of rats. Arrows represent exposure to a reinstatement stimulus or administration of drugs

The group of rats (*n* = 10 rats/group) that underwent a protocol of morphine self-administration and extinction training was used in reinstatement tests. Each rat underwent the reinstatement procedure in which either a morphine-associated cue or morphine solely (2.5–10 mg/kg, *ip*) was presented. The order of morphine injections was balanced according to a Latin square design, and the test sessions were separated by at least two to three baseline days of morphine self-administration (Fig. 1).

*Experiment 2: The effect of acute administration of DSF or NEP on morphine self-administration*

In pharmacological studies, the new cohort of rats divided into two separate groups and trained to self-administer morphine were acutely pretreated with either DSF (12.5–50 mg/kg; n = 9), or NEP (12.5–50 mg/kg; n = 9), or the corresponding vehicles 120 min before the self-administration sessions. The order of injections was balanced according to a Latin square design, and the test sessions were separated by at least two to three baseline days of morphine self-administration (Fig. 1).

*Experiment 3: The effect of repeated administration of DSF or NEP on extinction training and reinstatement of drug-seeking behavior*

A separate cohort of rats trained to self-administered morphine was divided into separate groups and pretreated with DSF (50 mg/kg; *n* = 11), NEP (25 mg/kg; *n* = 8) or the corresponding vehicles (control groups; *n* = 7 and 8) 120 min before the extinction session for 14 sequential days. The responses on active and inactive lever presses were compared to the control group. Following extinction training, morphine (5 mg/kg, *ip*) or the morphine-associated cue-induced reinstatement was tested in the same animals. The dose of DSF or NEP was selected based on the acute drug effect and previous research [13, 14, 24] (Fig. 1).

*Experiment 4: The effect of acute administration of DSF or NEP on reinstatement of drug-seeking behavior*

Other cohorts of rats with extinguished self-administration and divide to separated groups (Fig. 1) were used in the reinstatement tests. The rats were subjected to acute pretreatment with DSF (6.25–25 mg/kg; *n* = 10), NEP (6.25–25 mg/kg; *n* = 10) or the corresponding vehicles 120 min prior to commencement of the reinstatement sessions. Only one reinstatement protocol was applied to each rat, involving either a morphine infusion (5 mg/kg, *ip*) or presentation of a morphine-associated cue. A Latin square design was employed to set the injection order. Each animal received a drug combination in a randomized order in three to four reinstatement tests. Two to three extinction sessions at the minimum were run to separate each test session (Fig. 1).

#### Locomotor activity

The locomotor activity was recorded in Opto-Varimex cages (Columbus Instruments, Columbus, OH, USA) as described previously [25, 26]. Separate groups of naive rats were tested for the effect of acute or repeated (14 days) administration of DSF (6.25–50 mg/kg, *n* = 5–7 rats/groups) or NEP (6.25–50 mg/kg, *n* = 7–10 rats/groups) on locomotor activation. The naive rats were habituated 2 days/45 min before the administration of the drug. On the test day, animals were put into experimental cages and the locomotor activity was recorded for 120 min.

### Biochemical analyses

#### Surgical procedures

Guide cannulae (MAB 4; AgnTho's, Stockholm, Sweden) aimed at the nucleus accumbens, including the shell and core [anteroposterior: + 1.7 mm; mediolateral: + 1.0 mm; dorsoventral: − 5.8 mm] [27], were stereotaxically implanted as described previously [28]. To fix the guide cannulae to the rat skull, two miniature stainless steel screws and dental acrylic cement were used. Additionally, the guide cannulae was protected by obturators, which were maintained till the microdialysis test. After the surgery, all rats were allowed an 8-day recovery period.

#### Microdialysis procedures

On the test day, microdialysis probes (MAB 4, membrane with a molecular weight 6‐kDa cut‐off, 2‐mm length, 0.24‐mm outer diameter, AgnTho's AB, Lidingö, Sweden) were inserted into the guide cannulae (with the active membrane of internal cannulae extending 2 mm beyond the end of the guide cannulae) after obturator removal. Rats were then put into the experimental chambers and probes were connected to the liquid swivel (Instech, Plymouth Meeting, PA, USA) mounted on a counterbalanced arm at the top of a chamber and connected by polyethylene tubing (OD 0.68‐mm; AgnTho's) to a microinfusion pump (CMA/Microdialysis, Dalvägen, Sweden). Microdialysis probes were perfused with an artificial cerebrospinal fluid (in mM: NaCl 147, KCl 4.0, MgCl_2_ 1.0_,_ CaCl_2_ 2.2, pH 7.4) at a constant flow rate (2 μl/minute). The collection of dialysate samples commenced 80 min after the onset of perfusion to achieve stable dialysis neurotransmitters levels and perfusates. Following the collection of four dialysate samples (every 24 min thereafter, time period from − 96 to 0 min), used to determine spontaneous neurotransmitter levels (i.e. baseline samples), subsequent five samples were collected during the 2‐hour session (time period from 0 to 120 min). All the samples were immediately frozen and stored at − 20 °C until the later determination of extracellular neurotransmitter levels. DSF (12.5–50 mg/kg; *n* = 4–7), NEP (12.5–25 mg/kg; *n* = 4–6) and the corresponding vehicles (*n* = 7–9) were administered acutely during the microdialysis test or for 13 sequential days before and once during the microdialysis test (14 days of administration in total).

#### Measurement of dopamine and its metabolites

The levels of extracellular dopamine, 3,4-dihydroxyphenylacetic acid (DOPAC) and homovanillic acid (HVA) in 10 uL dialysate were assessed by ultra-high-performance liquid chromatography (UHPLC) with coulochemical detection. The UHPLC Ultimate 3000 system Dionex (Thermo Scientific, Germering, Germany) was equipped with an ECD-3000 RS electrochemical detector, 6011 RS ultra coulometric analytical cell, WPS-3000 RS autosampler and Hypersil Gold analytical column 3 µm, 100 × 3 mm (Thermo Scientific, Waltham, MA, USA). The mobile phase consisted of 0.1 M KH_2_PO_4_, 0.5 mM EDTA, 80 mg/l sodium 1-octanosulfonate and 4% methanol; adjusted to pH = 4.0 with 85% H_3_PO_4_. The flow rate of 0.6 ml/min and the column temperature of 30 °C were applied. The potentials of coulometric cell were: E1 =  − 50 mV, E2 =  + 350 mV. The external standard consisted of dopamine and DOPAC at concentrations of 50 ng/ml and HVA at a concentration of 100 ng/ml (Sigma). The chromatographic peaks were identified and quantized by comparison with the reference standard peaks. The Dionex Chromoleon 7 software (Thermo Scientific, Waltham, MA, USA) was used for data collection and analysis. The values were not corrected for in vitro probe recovery that amounted to approximately 15%. The limit of detection of dopamine, DOPAC and HVA was 0.5 pg/10 µL.

#### Histology

Immediately after the completion of the microdialysis experiment, rats were overdosed with sodium pentobarbital (morbital; 133.3 mg/ml; *ip*; Biowet, Puławy, Poland) and the brains were removed and stored in a 4% paraformalin (VWR Chemicals, Leuven, Belgium) solution for at least 3 days. Brains were cut on a cryostat and mounted on gel-coated glass slides. The placements of microdialysis probes were verified using a light microscope (PZO, Warszawa, Poland). Only data from rats with correctly placed probes within the nucleus accumbens, according to previously established guidelines, were included in statistical analyses. There was no necrosis distal to the track upon histological examination of the sections.

### Statistical analysis

In the case of all behavioral experiments, the data are represented as the means (± SEM). *Experiment 1:* during morphine self-administration and extinction training, the number of responses on the active and inactive levers was analyzed via a two-way repeated measures ANOVA with the factors *lever* (an active or inactive lever) and *session* as the repeated measure, or in the case of cue- or drug-reinstatement seeking behavior by a two-way ANOVA for factors *lever* (an active or inactive lever) and *phases* (self-administration, extinction and reinstatement of seeking behavior), respectively. *Experiment 2*: the effect of acute administration of DSF or NEP on morphine self-administration was analyzed by a one-way ANOVA for number of morphine infusions or a two-way ANOVA for factors *pretreatment* × *lever. Experiment 3*: the effect of repeated drug administration during extinction training and reinstatement seeking behavior was assessed using a three-way repeated measures ANOVA with the factors *pretreatment* × *lever* × *session*, and a three-way ANOVA for the *pretreatment* × *reinstatement* × *lever* interaction*. Experiment 4*: the effects of acute administration of DBH inhibitors on the reinstatement of seeking behavior were analyzed using a two-way ANOVA with factors *phases* × *lever* or *pretreatment* × *lever.* In experiments 1–4 (see above), post hoc comparisons were performed using a Newman–Keuls’ test for the significant effects identified by ANOVAs. Data for total locomotor activity were analyzed using a Student’s unpaired *t*-test, two-tailed for repeated administration, or one-way ANOVA with Dunnett’s post hoc for acute administration.

The results of the biochemical experiment were analyzed using a Student’s unpaired *t*-test, two-tailed for the basal level of dopamine and its metabolites or a two-way repeated measures ANOVA with *pretreatment* × *session* factors for the effect of acute or repeated administration of DSF or NEP.

Analyses in each behavioral and biochemical experiments were performed using the Statistica v.13 software (StatSoft Polska, Kraków, Polska). *p* < 0.05 was considered statistically significant. Graphs/figures were produced using the SigmaPlot v.12 and v.14 (Systat Software, San Jose, CA, USA) or GraphPad Prisma v. 8.1.1. (GraphPad Sofware Inc., La Jolla, CA, USA) software.

## Results

### Morphine self-administration and reinstatement of drug-seeking

In all experiments, rats acquired morphine self-administration and showed stable responses on the levers during the last three self-administration maintenance sessions with an acquisition criterion allowing variation in the number of active presses amounting to less than 15%.

#### Morphine self-administration (Experiment 1)

**Fig. 2:**
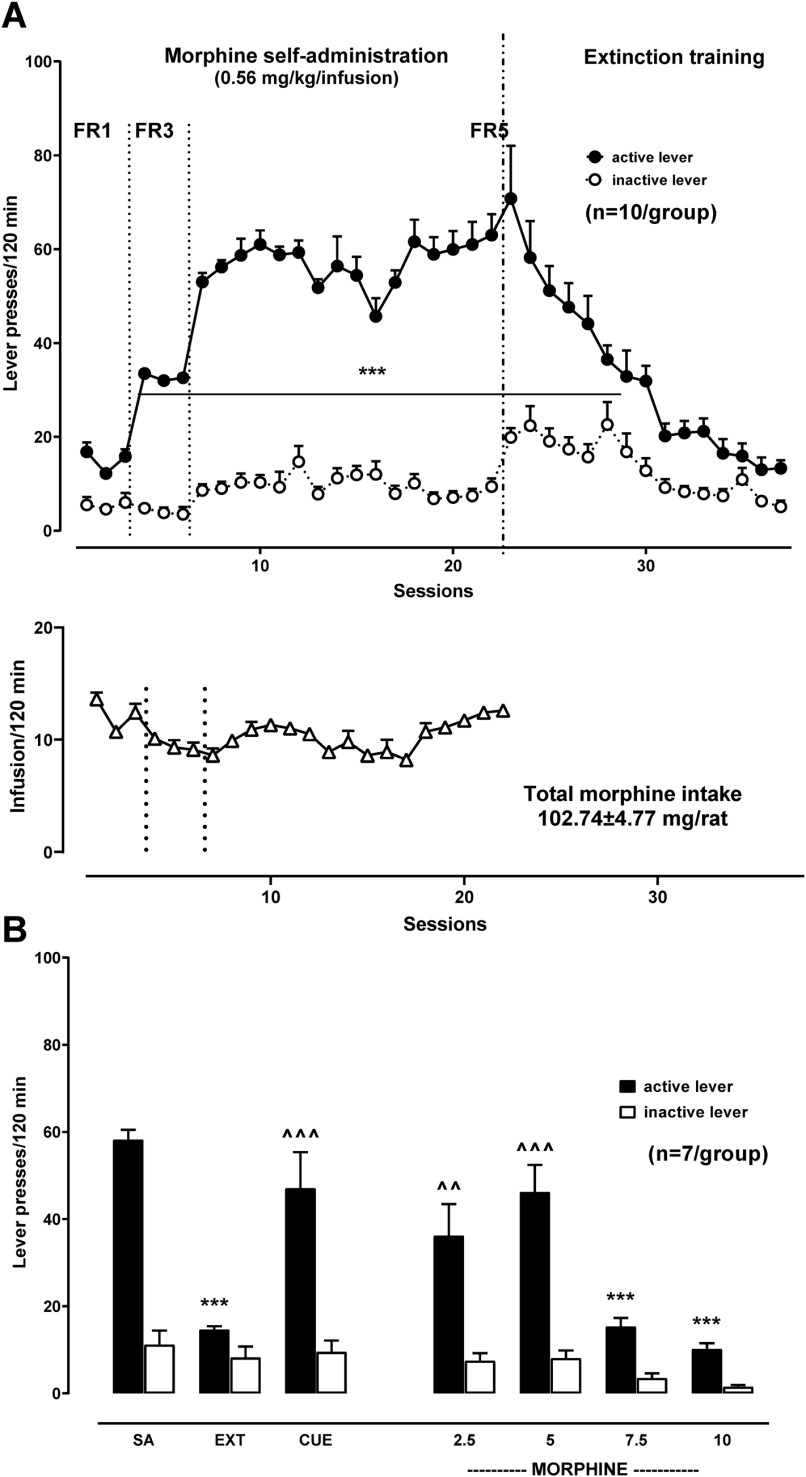
Morphine (0.56 mg/kg/infusion) self-administration (SA) under increasing schedule of reinforcement (FR1-5), extinction training (EXT) (panel **A** and **B**) and reinstatement seeking-behavior induced by a conditional stimulus associated previously with drug-taking (CUE; light + tones) and a non-conditional (morphine 2.5–10 mg/kg, ip) (panel **B** stimulus in rats. The number of active, inactive lever responses and infusions are expressed as means (± SEM) of data from 7 to 10 rats/group (the number of rats on the graph). The results were evaluated statistically using a two-way ANOVA with or without repeated measures, with Newman–Keuls’ post hoc. ***p < 0.001 versus SA-active lever; ^^*p*< 0.01, ^^^*p* < 0.001 versus EXT-active lever

As demonstrated in Fig. 2A, during 22-day self-administration, the number of active lever-responses increased between consecutive sessions, and when saline was substituted for morphine, a progressive drop in lever responses was seen over 15-day extinction training. A two-way ANOVA for repeated measures indicated a significant effect for the session [*F*_36,648_ = 24.70, *p* = 0.00002], lever [*F*_2,18_ = 243.49, *p* = 0.00002] and session × lever interaction [*F*_36,648_ = 16.41, *p* = 0.00002], while the Newman–Keuls’ post hoc analyses revealed that rats responded more on the active lever than the inactive lever from the 3rd till 29th (7th extinction) experimental day. The total quantity of morphine taken by rats during 22-day morphine self-administration was 102.74 ± 4.77 mg/rat, with a daily number of infusions during the last 3 sessions of maintenance of morphine self-administration in two trained groups ranging between 11.7 ± 0.40 and 12.60 ± 0.54. Similar results were observed for experiments (2–4) of morphine self-administration.

#### Morphine and morphine-associated cue reinstatement of seeking behavior (Experiment 1)

Reinstatement of seeking behavior induced by a cue or treatment with morphine (2.5–10 mg/kg) given before the start of the test session resulted in changes in the number of lever-responses (Fig. 2B).

A two-way ANOVA indicated a significant effect for the reinstatement (cue) [*F*_2,36_ = 7.82, *p* = 0.0015], lever [*F*_1,36_ = 50.09, *p* = 0.0002] and reinstatement (cue) × lever interaction [*F*_2,36_ = 3.71, *p* = 0.0342]. Reinstatement of seeking behavior induced by a cue associated with morphine self-administration increased of active lever-responses (p < 0.001), without changes in inactive lever-responses between experimental phases.

Treatment with morphine induced a significant effect for the reinstatement (drug) [*F*_5,72_ = 15.81, *p* = 0.0002], lever [*F*_1,72_ = 106.00, *p* = 0.0002] and reinstatement (drug) × lever interaction [*F*_5,72_ = 5.55, *p* = 0.0002] in rats that underwent morphine self-administration and extinction training. A Newman–Keuls’ post hoc test indicated significant increases of active lever-responses in the extinguished rats treated with the doses of 2.5 and 5 mg/kg (*p* < 0.01 and 0.001, respectively).

#### The effect of acute treatment with DSF or NEP on morphine self-administration (Experiment 2)

**Fig. 3:**
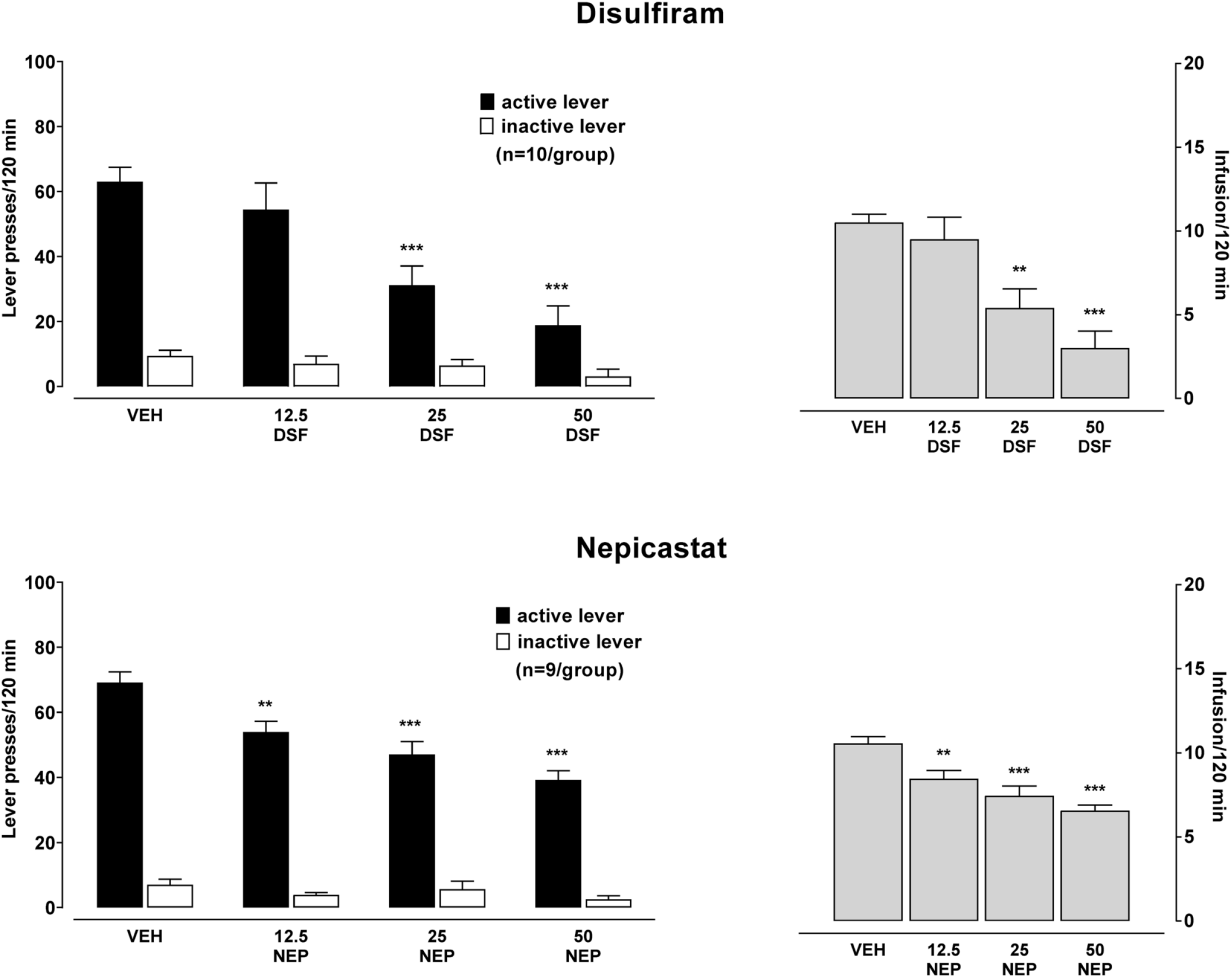
The effect of acute pre‐treatment with disulfiram (DSF, 12.5–50 mg/kg, ip, top panel), or nepicastat (NEP, 12.5–50 mg/kg, ip, bottom panel) or the corresponding vehicle (VEH) on active and inactive lever responses and on infusion during morphine (0.56 mg/kg/infusion) self-administration in rats. All bars represent the means (± SEM) of the data from 8 to 9 rats/group the (number of rats above bar). The results were evaluated statistically using a one- or two-way ANOVA with Newman–Keuls’ post hoc. **p < 0.01, ***p *p* 0.001 versus VEH-active lever or VEH

The effects of acute administration of DSF (12.5–50 mg/kg) and NEP (12.5–50 mg/kg) on active and inactive lever presses and the number of morphine infusions are shown in Fig. 3. A two-way ANOVA revealed significant effects for the pretreatment × lever interaction for DSF and NEP [*F*_3,72_ = 7.47, *p* = 0.0002 and *F*_3,64_ = 8.91, *p* = 0.00005, respectively]. The number of active lever-responses for morphine administration was suppressed in the rats treated with 25 and 50 mg/kg of DSF (*p* < 0.001) and with all used doses of NEP (p*p*< 0.01–0.001). Treatment with DSF or NEP given acutely before the start of the test session resulted in a dose-related reduction in the number of morphine infusions [a one-way ANOVA: *F*_3,36_ = 11.29, *p* = 0.00002 and *F*_3,32_ = 13.55, *p* = 0.00001]. A Newman–Keuls’ post hoc test indicated a significant reduction in the number of infusions in the rats treated with 25 and 50 mg/kg DSF (*p* < 0.01–0.001) and 12.5–50 mg/kg NEP (*p* < 0.01–0.001).

#### The effect of repeated administration of DSF or NEP on extinction training and reinstatement of drug-seeking behavior (Experiment 3)

After 21 days of morphine self-administration, an extinction training was introduced to all animals, without the drug or a drug-associated cue.

**Fig. 4:**
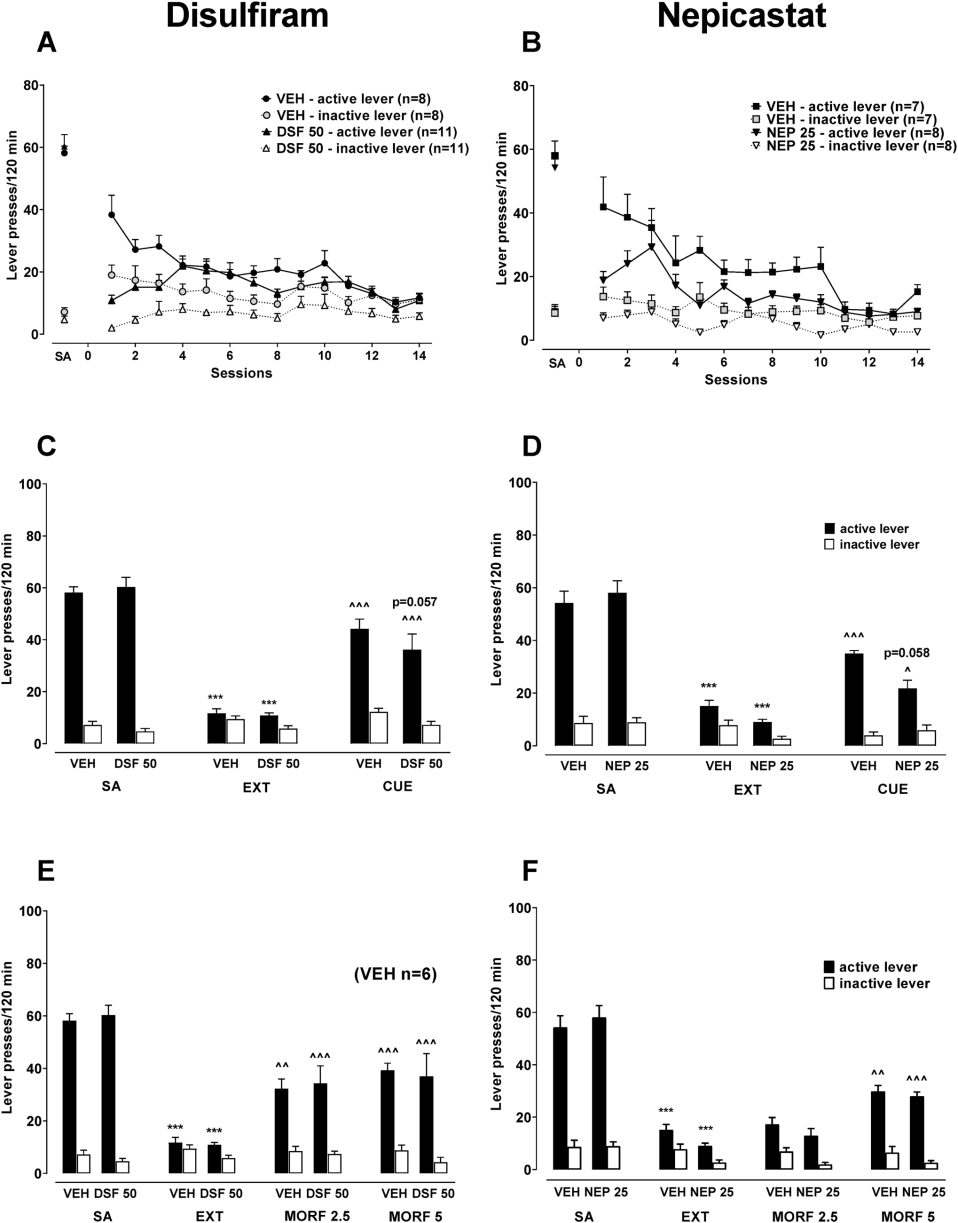
The effect of repeated pre‐treatment with disulfiram (DSF, 50 mg/kg, ip, panel **A**), nepicastat (NEP, 25 mg/kg, ip, panel **B**) or the corresponding vehicle (VEH) on active and inactive lever responses during extinction training (EXT) in rats previously self-administering morphine (SA) and their effect on reinstatement seeking-behavior induced by a conditional stimulus associated previously with drug-taking (CUE; light + tones, **C**, **D**) and a non-conditional (morphine 2.5–5 mg/kg, ip, **E**, **F**) stimulus in rats. All bars represent the means (± SEM) of the data from 7 to 11 rats/group (number of rats above bar). The results were evaluated statistically using a two-way repeated measures ANOVA with Newman–Keuls’ post hoc. ***p < 0.001 versus corresponding SA group-active lever, ^*p* < 0.05, ^^*p* < 0.01, ^^^ *p*< 0.001 versus corresponding EXT group-active lever

A three-way ANOVA did not indicate any effect for the session × pretreatment × lever interaction [*F*_13,442_ = 1.43, *p* = 0.1401 and *F*_13,338_ = 0.95, *p* = 0.5044] between groups that underwent repeated administration of vehicle, DSF (50 mg/kg) or NEP (25 mg/kg) (Fig. 4B) and during extinction training the reduction in active lever presses was observed (*p* < 0.001), as compared to the last morphine self-administration session in all experimental groups (Fig. 4).

##### Reinstatement of cue-induced seeking behavior

After 14-daily administration of vehicle or DSF (50 mg/kg), during the extinction-training period, rats were tested for the response reinstatement induced by a morphine-associated cue (Fig. 4C). As shown by a three-way ANOVA for the pretreatment × reinstatement (cue) × lever interaction [*F*_2,100_ = 0.32, *p* = 0.7266], reinstatement of seeking behavior induced by a cue was relevant following either vehicle or DSF pretreatment (at least *p* < 0.05), but did not reveal a significant difference between groups treated with vehicle or DSF, suggesting that rats responded similarly to the cue. However, during the cue-induced reinstatement in rats treated with DSF attenuation of active lever-responses compared to the control group (*p* = 0.0573) was observed.

Similarly, a three-way ANOVA for the pretreatment × reinstatement (cue) × lever interaction [*F*_2,78_ = 2.76, *p* = 0.0695] did not indicate a significant effect of the repeated administration of vehicle or NEP (25 mg/kg) during the extinction training. Further, the cue-induced reinstatement was similar (at least *p* < 0.05) (Fig. 4). However, an analysis of active lever presses during reinstatement of seeking behavior revealed attenuation of active lever-responses in animals treated with NEP, respectively (*p* = 0.0580).

##### Reinstatement of morphine-induced seeking behavior

A three-way ANOVA for factors pretreatment, reinstatement (drug), and lever demonstrated that repeated treatment with DSF (50 mg/kg) or NEP (25 mg/kg) during extinction training did not change increased lever presses (at least *p* < 0.01) noted during reinstatement [*F*_3,120_ = 0.002, *p* = 0.9999 and *F*_3,100_ = 0.27, *p* = 0.8450, respectively] (Fig. 2E, F).

#### The effect of acute administration of DSF or NEP on reinstatement of morphine-seeking behavior (Experiment 5)

##### Reinstatement of cue-induced seeking behavior:

**Fig. 5:**
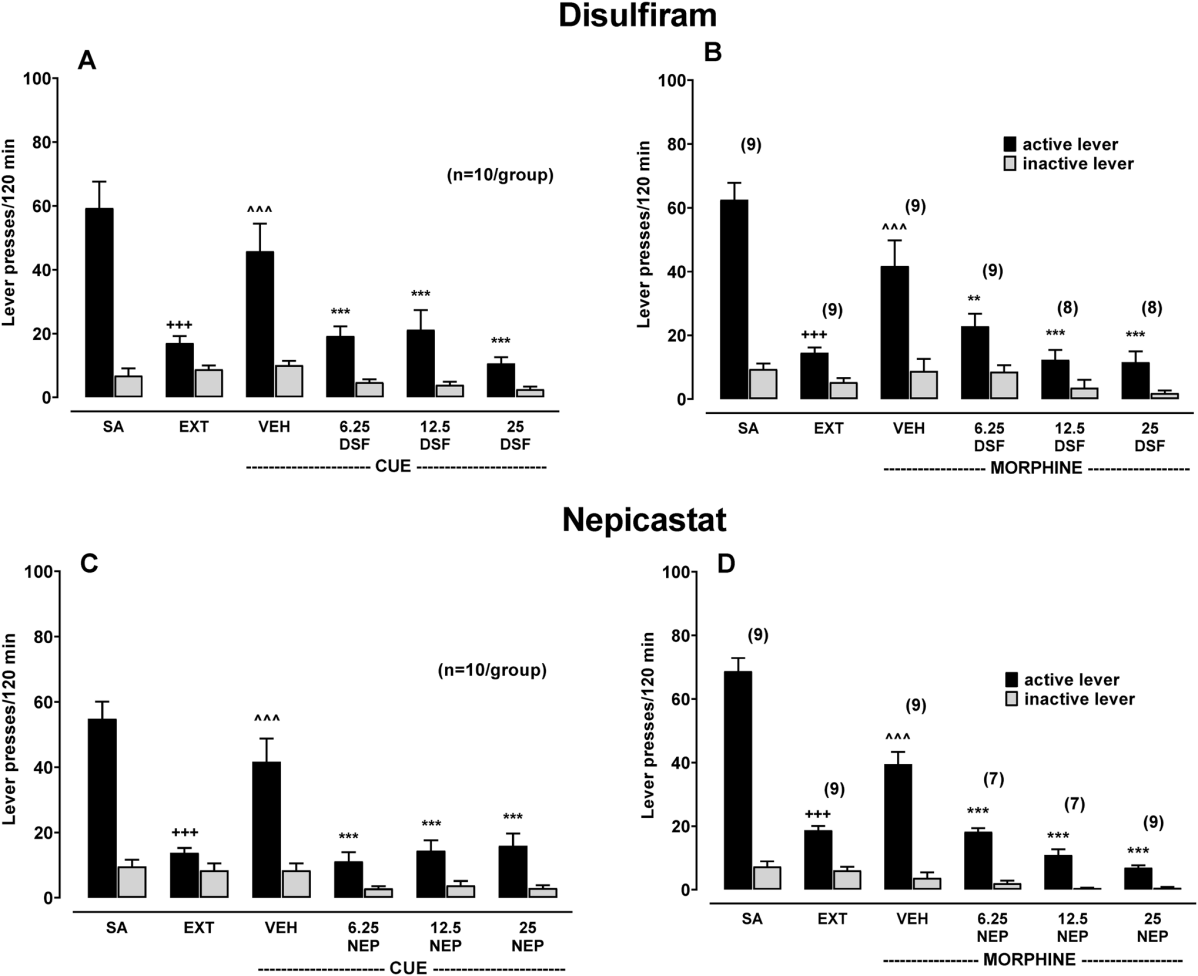
The effect of acute pre‐treatment with disulfiram (DSF, 6.25–25 mg/kg, ip, panels **A**, **B**), nepicastat (NEP, 6.25–25 mg/kg, ip, panels **C**, **D**) or the corresponding vehicle (VEH) on active and inactive lever responses on reinstatement seeking-behavior induced by a conditional stimulus associated previously with drug-taking (CUE; light + tones) and a non-conditional (morphine 5 mg/kg, ip) stimulus in extinguished (EXT) to morphine self-administrating (SA) rats. All bars represent the means (± SEM) of the data from 7 to 10 rats/group (number of rats above bar). The results were evaluated statistically using a two-way ANOVA with Newman–Keuls’ post hoc. +++*p* < 0.001 versus SA-active lever; ^^^*p* < 0.001 versus EXT-active lever, **p < 0.01, ***p < 0.001 versus VEH

The 14-day extinction training led to the reduction in active lever presses (*p* < 0.001), more than inactive lever presses, compared to the last morphine self-administration session in both experimental groups [a two-way ANOVA: *F*_1,36_ = 19.47, *p* = 0.0001 and *F*_1,36_ = 27.93, *p* = 0.0001] (Fig. 5C).

Regarding the cue-induced seeking behavior, measured as increases of active lever-responses (*p* < 0.001), a two-way ANOVA revealed an effect for the cue-induced reinstatement of seeking behavior in both experimental groups [*F*_1,36_ = 9.06, *p* = 0.0048 and *F*_1,36_ = 7.93, *p* = 0.0078] (Fig. 5A, C).

When DSF (6.25–25 mg/kg) was administered 2 h before placing rats in the experimental cage, a marked reduction in the number of active lever-responses induced by the cue was observed [a two-way ANOVA: *F*_3,72_ = 4.26, *p* = 0.0079]. A Newman–Keuls’ post hoc test demonstrated a reduction in active lever presses in rats treated with all used doses of DSF (*p* < 0.001) (Fig. 5A), in comparison to vehicle-treated rats.

Similarly, treatment with NEP (6.25–25 mg/kg) reduced the cue-induced reinstatement. A two-way ANOVA indicated a significant effect for the pretreatment × lever interaction [*F*_3,72_ = 3.94, *p* = 0.0116]. A Newman–Keuls’ post hoc test indicated decreases in active lever responses (*p* < 0.001) for all used doses of NEP during the reinstatement of seeking behavior induced by a morphine-associated cue (Fig. 5C).

#### Reinstatement of the drug-induced seeking behavior

All rats were extinguished after 14-day experimental procedure [a two-way ANOVA: *F*_1,32_ = 52.06, *p* < 0.0000 and *F*_1,32_ = 100.91, *p* < 0.0000], and a significant reduction of the active lever-presses (*p* < 0.001) during the last session of extinction training compared to the last session of morphine self-administration was indicated (Fig. 5B, D).

The administration of morphine (5 mg/kg) reinstated rats' drug-seeking behaviors in both experimental groups [a two-way ANOVA: *F*_1,32_ = 6.58, *p* = 0.0152 and *F*_1,32_ = 24.46, *p* = 0.00002]. The number of active lever-responses during the reinstatement session was raised substantially (*p* < 0.001) (Fig. 5B, D).

A two-way ANOVA indicated a significant effect of treatment with DSF (6.25–25 mg/kg) on the number of lever-responses during morphine-induced reinstatement [*F*_3,60_ = 3.73, *p* = 0.0158]. In fact, the number of active lever-responses was reduced in the rat groups treated with all used doses of DSF (*p* < 0.05–0.001) (Fig. 5B).

A reduction of reinstatement seeking behavior induced by re-treatment with morphine (5 mg/kg) was demonstrated after NEP (6.25–25 mg/kg) [a two-way ANOVA for factors pretreatment × lever: *F*_3,56_ = 25.82, *p* < 0.0000]. A post hoc test revealed a marked reduction in the active lever presses in rats treated with all doses of NEP (*p* < 0.001) (Fig. 5D).

### Locomotor activity

**Fig. 6:**
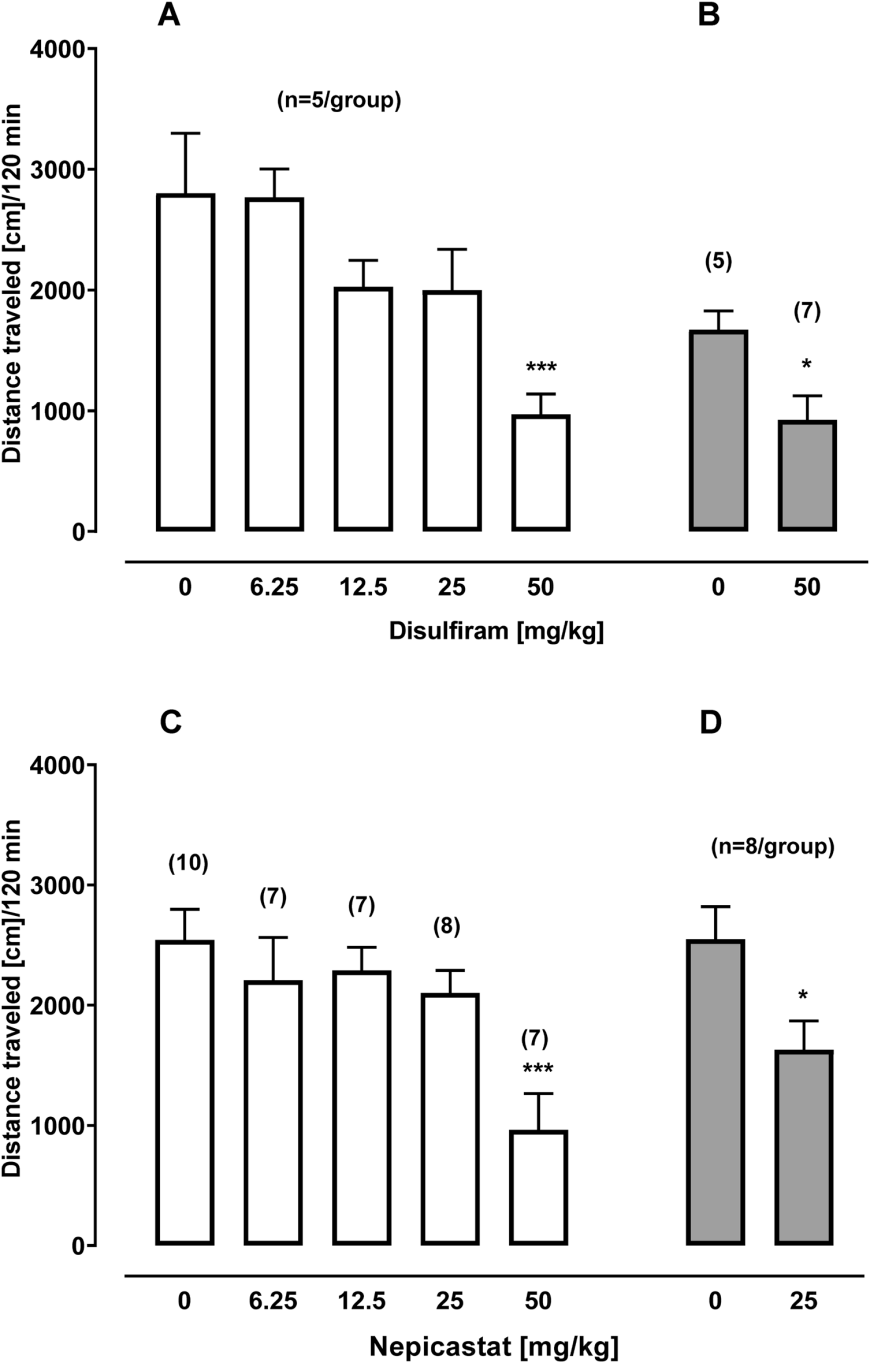
The effect of acute (**A**, **C**) and repeated (**B**, **D**) administration of disulfiram (6.25–50 mg/kg; ip, n = 5–7) and nepicastat (6.25–50 mg/kg; ip, n = 7–10) on locomotor activity (mean ± SEM; number of rats above bar). *p < 0.05, ***p< 0.001 versus 0. The results were evaluated statistically using a Student’s unpaired t-test, two-tailed or one-way ANOVA with Dunnett’s post hoc

When DSF or NEP was administered acutely 2 h before transferring naive rats into the locomotor activity cage, it produced a marked change in locomotor activity [*F*_4,25_ = 8.52; *p* = 0.0002 and *F*_4,34_ = 5.84; *p* = 0.0011, respectively] (Fig. 6C). In comparison to vehicle-treated rats, the distanced traveled was reduced by ca. 65% in rats given 50 mg/kg of either DSF or NEP. Similarly, a reduction by ca. 45% and 36% [*t*_10_ = 2.41; *p* = 0.0364 and *t*_14_ = 2.53; *p* = 0.0241] in locomotor activity was recorded after repeated (14 days) treatment with 50 mg/kg DSF or 25 mg/kg NEP (Fig. 6B, D).

### The effect of acute and repeated administration of DSF or NEP on the extracellular level of dopamine and its metabolites in the nucleus accumbens

**Fig. 7:**
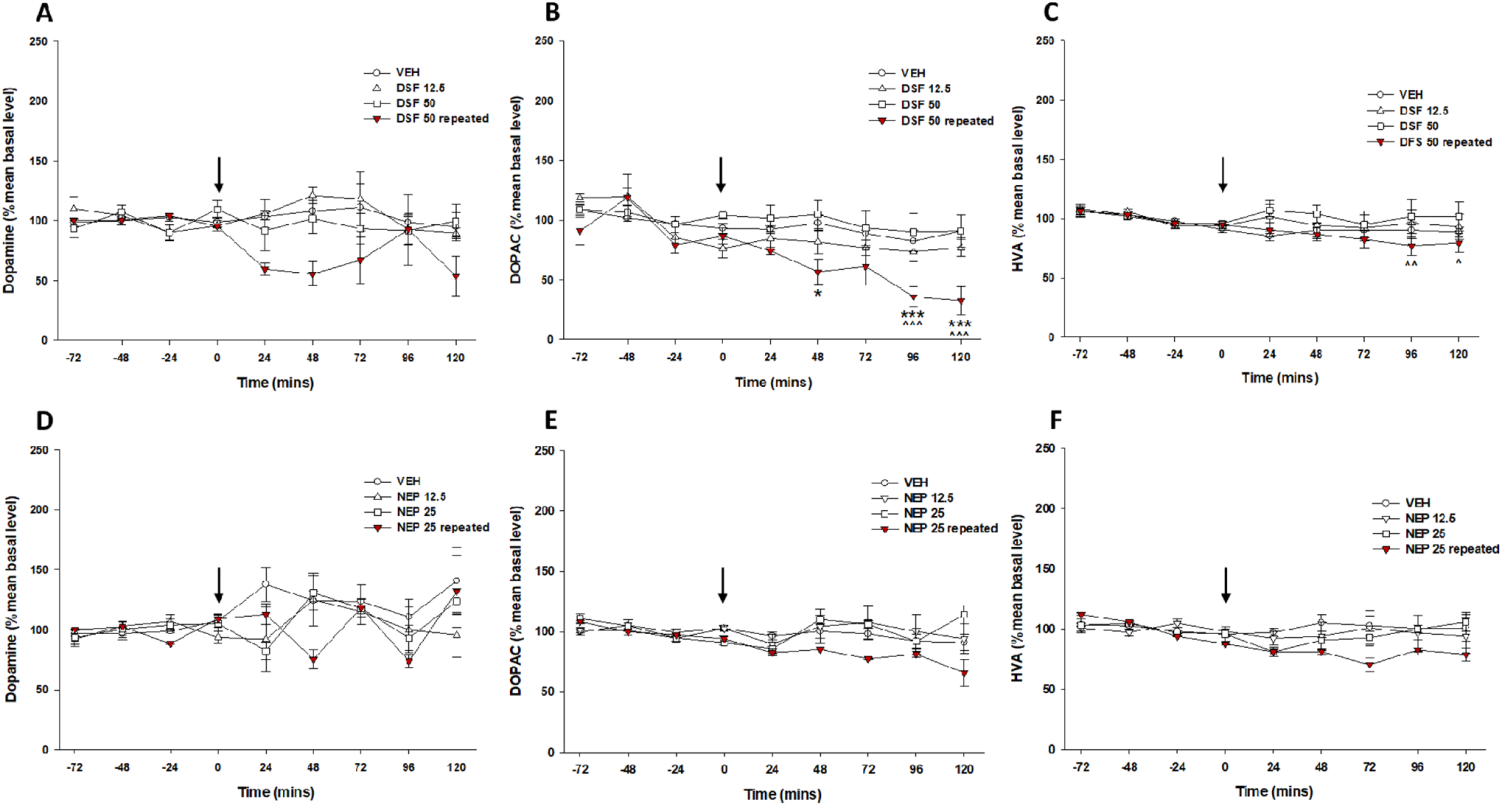
The effect of acute and repeated pretreatment with disulfiram (DSF, 12.5–50 mg/kg, ip, panels **A**–**C**) or nepicastat (NEP, 12.5–25 mg/kg, ip; panels **D**–**F**) or the corresponding vehicle (VEH) on the extracellular dopamine and its metabolites DOPAC and HVA levels in the nucleus accumbens. Arrows represent administration of DSF, NEP or VEH. Data are expressed as a percentage of mean basal level and shown as the mean ± SEM of 4–9 rats. The results were evaluated statistically using a two-way repeated measures ANOVA with Newman–Keuls’ post hoc. *p < 0.05, ***p  < 0.001 versus corresponding VEH, ^*p* < 0.05, ^^*p* < 0.01, ^^^*p* < 0.001 versus 0 min

After the surgery and recovery period, the rats experimentally naïve were tested to measure variations in the level of extracellular dopamine and its metabolites, DOPAC and HVA using microdialysis in the nucleus accumbens (Table 1 and Fig. 7).

**Table 1:** The effect of 13-day administration of disulfiram (DSF, 50 mg/kg; ip,* n* = 4), nepicastat (NEP, 25 mg/kg; ip, n = 4) or the corresponding vehicle (VEH, ip, n = 7–9) on the extracellular basal levels of dopamine (DA) and its metabolites, DOPAC and HVA, in the nucleus accumbens

	DA [pg/10 uL]	DOPAC [pg/10 uL]	DOPAC/DA ratio	HVA [pg/10 uL]	HVA/DA ratio
VEH	3.89 ± 1.14	777.86 ± 128.81	256.91 ± 49.13	509.16 ± 84.32	177.70 ± 42.25
DSF 50 mg/kg	1.49 ± 0.21	248.31 ± 35.70*	175.67 ± 31.68	525.75 ± 71.21	369.09 ± 58.63*
	t9 = 1.54, p = 0.16	t9 = 3.00, p < 0.05	t9 = 1.15, p = 0.27	t9 = − 0.13, p = 0.90	t9 = − 2.69, p < 0.05
VEH	2.69 ± 0.44	969.35 ± 147,09	396.62 ± 45.81	551,07 ± 73,6	252.08 ± 48.44
NEP 25 mg/kg	1.21 ± 0.07	701.54 ± 93,54	590.42 ± 0.10	591.41 ± 90.57	494.49 ± 38.42*
	t11 = 2.16, p = 0.05	t11 = 1.15, p = 0.28	t11 = − 2.14, p = 0.06	t11 = − 0.36, p = 0.72	t11 = − 3.10, p = < 0.05

All data expressed the means (± SEM) of the data from 4 to 9 rats/group. The results were evaluated statistically using a Student’s unpaired t-test, two-tailed. *p < 0.05 versus VEH

DSF administered at a dose of 50 mg/kg *ip* for 13 days resulted in a reduction (and a tendency to decrease) basal concentration of dopamine and DOPAC (down to 38% and 32% of the vehicle, respectively), but not HVA compared to the corresponding vehicle (Table 1). Repeated injections of DSF did not significantly affect the DOPAC/DA ratio, but significantly increased the HVA/DA ratio (up to 208% vehicle value).

Pretreatment with NEP at a dose of 25 mg/kg *ip* for 13 days decreased the basal concentrations of dopamine and DOPAC (down to 45% and 72%, respectively), and lacked any significant impact on the DOPAC/DA ratio. The HVA level remained unchanged, however, the HVA/DA ratio measured in the tested structure was significantly increased (up to 196% of the vehicle) (Table 1).

A two-way repeated measures ANOVA did not indicate any significant effect of acute DSF treatment with doses of 12.5 and 50 mg/kg on factors: pretreatment × session for any of the measured parameters, dopamine, DOPAC and HVA [*F*_10,80_ = 0.93, *p* = 0.5103; *F*_10,80_ = 0.22, *p* = 0.9933; *F*_10,80_ = 0.42, *p* = 0.9282, respectively] (Fig. 7A–C).

The repeated treatment with DSF at a dose of 50 mg/kg did not significantly change the dopamine level during microdialysis [pretreatment × session interaction: *F*_5,45_ = 2.01, *p* = 0.0948](Fig. 7A). A reduction in dopamine level was observed during that time but the overall analysis indicated that this effect was insignificant. Instead, a repeated measures ANOVA revealed a significant difference in the level of DOPAC and HVA [*F*_5,45_ = 3.11, *p* = 0.0170; *F*_5,45_ = 3.27, *p* = 0.0132, respectively]. A Newman–Keuls’ post hoc test demonstrated a reduction of the DOPAC level compared to the corresponding vehicle (*p* < 0.05–0.001) and basal level (*p* < 0.001) (Fig. 7B). Similarly, a Newman–Keuls’ post hoc test performed for the HVA level indicated a reduction for the repeated DSF administration (*p* < 0.01) between the first and last measurement (Fig. 7C).

Acute administration of NEP at doses of 12.5 and 25 mg/kg during microdialysis test session did not change the dopamine, DOPAC or HVA levels [*F*_10,85_ = 1.52, *p* = 0.1462; *F*_10,85_ = 1.63, *p* = 0.1133; *F*_10,85_ = 1.17, *p* = 0.3207, respectively] (Fig. 7D–F).

Similarly, a repeated measures ANOVA did not indicate any significant effect of repeated administration of NEP in a dose of 25 mg/kg on the measured parameters during microdialysis: dopamine, DOPAC and HVA [*F*_5,55_ = 1.75, *p* = 0.1384; *F*_5,55_ = 1.52, *p* = 0.1999; *F*_5,55_ = 1.53, *p* = 0.1949, respectively] (Fig. 7D–F).

## Discussion

### Morphine self-administration, extinction training, and reinstatement of the drug-seeking behavior

In the present study during the 22-day period of morphine self-administration, the animals maintained a stable level of morphine intake (ca. 6 mg of the drug in the last session of self-administration). Replacing morphine with saline caused a robust reduction of active lever presses, which was extinguished after 14 days of exposure to the experimental cage during extinction training sessions. The presented data confirm earlier reports that morphine can serve as a positive reinforcer in rats, and the pattern lever presses during maintenance of self-administration and extinction training stayed in line with a previous study [29–36].

Reinstatement of seeking-behavior in an animal model has been consistently described in the literature as a representative model of the propensity to relapse in humans [37]. Presentation of a conditional stimulus previously associated with morphine self-administration and a non-conditional stimulus (morphine, *ip*) resulted in relevant changes in lever presses in rats with extinguished seeking-behavior. Reinforcement behavioral reaction was related to an increase of active lever presses, but not inactive, for both stimuli. During morphine-induced reinstatement, the active lever responses increased more markedly after exposure to doses 2.5 and 5 mg/kg than 7.5 and 10 mg/kg. In the case of higher morphine doses, the lower active lever presses were associated with inactive lever presses (trend to reduce), which means that the behavioral response was sedation in general. Previous studies also show a robust rise in the number of responses reinforced by a conditional and non-conditional stimulus during reinstatement of seeking behavior [30, 32, 36, 38, 39].

#### The effect of acute or repeated treatment with DSF or NEP on morphine self-administration, extinction training, and reinstatement seeking behavior

Another notable finding presented in the study is that both investigated DBH inhibitors reduced morphine intake and behavioral responses connected with active lever presses during self-administration of morphine in rats. Moreover, the present findings have shown that DSF and NEP administered acutely before the reinstatement test session consistently attenuated the reinforcing effects of morphine and a cue associated previously with drug-taking. However, 2-h pretreatment with DSF or NEP in the highest doses (50 mg/kg), markedly suppressed spontaneous locomotor activity, suggesting that these doses may have contributed, at least in part, to the suppressant effect of DBH inhibitors on morphine self-administration. Nevertheless, lower doses of DSF and NEP prior to morphine self-administration effectively reduced active lever-responding related to morphine reinforcement in rats. The above effects were specific as both DBH inhibitors neither changed locomotor activity when given alone to naïve rats nor altered the inactive lever pressing in the morphine self-administration model. In the study with repeated administration of DSF (50 mg/kg) and NEP (25 mg/kg), neither drug modified inactive lever presses during extinction training in morphine-exposed rats and the reinstatement of seeking behavior was not changed, despite their potent reduction locomotor activity in naïve animals. So far available preclinical trials have focused only on psychostimulants and provide mixed support for the idea that DBH inhibitors show promise as a treatment for substance use disorder. Although cocaine and morphine have different pharmacological mechanisms of action, the repeated treatment of these drugs leads to a rise in dopamine levels and a reduction in basal glutamate levels in sub-cortical and/or cortical brain areas. Thus, early studies showed that DSF pretreatment suppresses psychostimulants-induced locomotor activity in rodents [40, 41]. Other studies did not confirm any positive effect of acute administration of DSF and NEP on responding to cocaine during the maintenance phase of self-administration under an FR1 schedule of responding [13], but DSF—at the limited range of doses—consistently attenuated the reinforcing effects of *d*-methamphetamine [42]. Nevertheless, both DBH inhibitors reduce breakpoint responding for cocaine on a progressive ratio schedule, thus suggest that DBH inhibition may selectively attenuate the positive reinforcing effects of psychostimulants under conditions that require high motivation [14]. Clinical research revealed that DBH inhibitors can result in a reduction of the positive subjective effects of several psychostimulants [43, 44]. Other previous research shows that two DBH inhibitors, DSF and NEP, administered acutely in rodent's attenuate reinstatement of cocaine-seeking induced by a drug-primed conditional cue, stress, or even yohimbine [13–15, 45]. Instead, Cooper and co-workers [46] reported that DSF failed to attenuate cocaine reinstatement in squirrel monkeys.

Neither DSF nor NEP administered for 14 days during extinction training influenced lever responding. In the reinstatement of drug-seeking behavior induced by a non-contingent stimulus procedure, intended to reproduce reinstatement in lever responses related previously with self-administration of morphine, controls, and animals chronically treated with DBH inhibitors was not different. Interestingly, reinstatement of drug-seeking behavior induced by a cue previously associated with the availability of the reward, demonstrated a trend to lower responses in groups that received repeated administration of DSF or NEP during the abstinence period.

The nucleus accumbens is involved in the reinstatement of drug-seeking behavior induced by conditional and non-conditional stimuli and its role is related to a dopamine release [47, 48]. In addition, the nucleus accumbens is found to be involved in the extinction of conditioned behavior, thought to be a process of new and active learning [49, 50]. The drugs of abuse induce a large release of dopamine in limbic areas, specifically in the nucleus causes feelings of pleasure. Consistently with previous observations, in control rats DSF and NEP administered acutely did not change the extracellular level of dopamine and its metabolites 2 h after treatment [17, 47]. In the present study, repeated, but not single, administration of DSF or NEP for several days to rats tended to reduce the extracellular basal levels of dopamine and DOPAC suggesting a diminished release of dopamine in the synaptic cleft and/or augment dopamine reuptake. In fact, a significant increase in the accumbal HVA/DA ratio, without an effect on dopamine metabolite HVA was observed in rats. Dopamine is broken down into inactive metabolites by a set of enzymes. The major intracellular enzymes involved in neurotransmitter degradation are monoamine oxidases which convert dopamine into the intermediate metabolite 3,4-dihydroxyphenylacetaldehyde, and then into DOPAC. The other major enzyme related to extracellular dopamine metabolism is catechol-O-methyltransferase which transforms the neurotransmitter into 3-methoxytyramine (3-MT). Finally, both DOPAC and 3-MT are converted into HVA [51]. Because DBH inhibitors directly influenced the level of dopamine this mechanism might belong to DSF and NEP reduction of the reinforcing effects of abused drugs. As found, both DBH inhibitors reduced extracellular noradrenaline in the prefrontal cortex and nucleus accumbens [13–15, 20, 46, 52]. Anatomical and functional relationships between the noradrenergic, dopaminergic, and glutamatergic systems influence the sensitivity of the mesolimbic reward system to substance use disorder. Since norepinephrine stimulates the drive of dopamine neurons, both directly (via the ventral tegmental area) and indirectly (via glutaminergic projections from the prefrontal cortex to the ventral tegmental area) [53], inhibition of DBH would reduce the stimulation of dopamine neurons and the extracellular accumbal dopamine levels. Additionally, it is well documented the loss of norepinephrine in the prefrontal cortex also attenuates the drug-induced dopamine release in the nucleus accumbens [44, 53].

Major disadvantages of the presented microdialysis results are concerned with the doses of DBH inhibitors, which not fully correlated with behavioral experiments of morphine self-administration, and limiting data to only one brain structure, where drugs of abuse induce a large release of dopamine in several limbic areas not only in nucleus accumbens. However, based on these data, it might be timidly suggested that both DSF and NEP control behavioral responses by an inhibitory action on noradrenaline synthesis and release in the nucleus accumbens, while present data did not exclude their effect on dopamine metabolism and release. Moreover, an increased release of dopamine instead of noradrenaline from noradrenergic terminals in the prefrontal cortex cannot be ruled out.

## Conclusions

A major outcome of this study is that DSF and NEP reduced morphine self-administration and reinstatement of drug-seeking behavior induced by both conditional and non-conditional stimulus. Repeated administration of both DBH inhibitors admittedly did not influence the extinguished seeking behavior but attenuated reinstatement of seeking behavior induced by a stimulus previously associated with morphine taking. Moreover, this study has provided evidence that DBH inhibitors induced changes in behavioral responses related to morphine. These preclinical results point to a significance of DBH inhibition as a potential pharmacotherapy against morphine abuse, though limitations, such as the biochemical analysis of only one structure and focus on male sex, indicate a need for further studies to prove the beneficial potential of DBH inhibitors.

Some evidence from randomized controlled trials showed the effectiveness of DSF treatment in people with cocaine dependence and alcohol users toward fewer dropouts from psychosocial treatment, reduced cocaine positive urine toxicology screens and a greater number of weeks of abstinence [12, 44, 52, 54]. Nevertheless, DSF has been founded to display poor effectiveness in opioid co-dependent subjects [54, 55]. Furthermore, current clinical trials do not suggest the use of DBH inhibitors in the therapy of OUD or alcohol dependent subjects exhibiting comorbid opioid-related problems. It needs to be determined whether higher potency DBH inhibitors, such as NEP, are efficacious in reducing not only cocaine but also opioid use in dually cocaine and opioid dependent individuals.
